# Deep Learning–based Diagnosis of Pulmonary Tuberculosis on Chest X-ray in the Emergency Department: A Retrospective Study

**DOI:** 10.1007/s10278-023-00952-4

**Published:** 2024-01-10

**Authors:** Chih-Hung Wang, Weishan Chang, Meng-Rui Lee, Joyce Tay, Cheng-Yi Wu, Meng-Che Wu, Holger R. Roth, Dong Yang, Can Zhao, Weichung Wang, Chien-Hua Huang

**Affiliations:** 1 Department of Emergency Medicine, College of Medicine, National Taiwan University, Taipei, Taiwan; 2 Department of Emergency Medicine, National Taiwan University Hospital, No. 7, Zhongshan S. Rd, Zhongzheng Dist., Taipei City, 100 Taiwan; 3 Department of Mathematics, National Taiwan University, Taipei, Taiwan; 4 Department of Internal Medicine, National Taiwan University Hospital, Taipei, Taiwan; 5 NVIDIA Corporation, Bethesda, MD USA; 6 Institute of Applied Mathematical Sciences, National Taiwan University, No. 1, Sec. 4, Roosevelt Rd., Taipei, 106 Taiwan

**Keywords:** Tuberculosis, Deep learning, Chest X-ray, Chest radiograph, Emergency medicine

## Abstract

Prompt and correct detection of pulmonary tuberculosis (PTB) is critical in preventing its spread. We aimed to develop a deep learning–based algorithm for detecting PTB on chest X-ray (CXRs) in the emergency department. This retrospective study included 3498 CXRs acquired from the National Taiwan University Hospital (NTUH). The images were chronologically split into a training dataset, NTUH-1519 (images acquired during the years 2015 to 2019; *n* = 2144), and a testing dataset, NTUH-20 (images acquired during the year 2020; *n* = 1354). Public databases, including the NIH ChestX-ray14 dataset (model training; 112,120 images), Montgomery County (model testing; 138 images), and Shenzhen (model testing; 662 images), were also used in model development. EfficientNetV2 was the basic architecture of the algorithm. Images from ChestX-ray14 were employed for pseudo-labelling to perform semi-supervised learning. The algorithm demonstrated excellent performance in detecting PTB (area under the receiver operating characteristic curve [AUC] 0.878, 95% confidence interval [CI] 0.854–0.900) in NTUH-20. The algorithm showed significantly better performance in posterior-anterior (PA) CXR (AUC 0.940, 95% CI 0.912–0.965, *p-value* < 0.001) compared with anterior–posterior (AUC 0.782, 95% CI 0.644–0.897) or portable anterior–posterior (AUC 0.869, 95% CI 0.814–0.918) CXR. The algorithm accurately detected cases of bacteriologically confirmed PTB (AUC 0.854, 95% CI 0.823–0.883). Finally, the algorithm tested favourably in Montgomery County (AUC 0.838, 95% CI 0.765–0.904) and Shenzhen (AUC 0.806, 95% CI 0.771–0.839). A deep learning–based algorithm could detect PTB on CXR with excellent performance, which may help shorten the interval between detection and airborne isolation for patients with PTB.

**Supplementary Information:**

The online version contains supplementary material available at 10.1007/s10278-023-00952-4.

## Introduction

### Background

There were estimated 10 million tuberculosis infections reported worldwide in the year 2020, with an estimated 1.3 million deaths due to tuberculosis [1]. Patients with active pulmonary tuberculosis (PTB) often make multiple emergency department (ED) visits before diagnosis [2]. Correct diagnosis in the ED serves an important role in public health by curbing the spread of PTB.

The systematic review by Harris et al. [3] indicated that deep learning (DL)–based algorithms had superior accuracy in diagnosing PTB on chest X-rays (CXRs). Harris et al. [3] also found that the potential risk of bias was common in the databases used to assess the derived algorithms in diagnosing PTB, which may lead to overestimated performance in previous studies. To avoid potential bias, Harris et al. [3] advocated that studies aimed to develop PTB-detecting algorithms should (1) describe how CXRs were selected for training and testing, (2) use CXRs from distinct databases for training and testing, and (3) assess the accuracy of the derived algorithm against a microbiologic reference standard.

### Importance

While prompt early diagnosis of PTB accompanied by airborne isolation procedures [4] is paramount to preventing nosocomial infections in overcrowded EDs [5], it is reported that fewer than half of newly diagnosed PTB patients are identified during their ED stay, and less than one-fifth of these patients are isolated in the ED [6]. This delay in diagnosis and isolation of PTB patients can pose threats to critically ill hospitalised patients and also to healthcare providers [7].

The diagnostic delays [8] have accompanied a decline in the reported prevalence of PTB [9], and emergency physicians may have become less familiar with the presentation of PTB [10]. However, PTB prevalence remains high in various socioeconomically disadvantaged populations [11]; these are often the same populations who may disproportionately rely on ED visits for health care [12]. Prompt diagnosis of PTB at EDs should remain a priority to ensure timely treatment and prevention of community outbreaks.

### Goals of This Investigation

CXR is key to the diagnosis of PTB, but the success of CXR as a screening and triage tool can be limited by high inter- and intra-reader variability and moderate specificity [13]. Therefore, in the current study, we aimed to develop and test a DL-based computer-aided diagnosis (CAD) algorithm for the detection of PTB by CXR in the ED.

## Materials and Methods

### Study Design and Setting

We conducted a retrospective study to develop a CAD algorithm for detecting PTB on CXRs and test its performance in the local population and public databases. This study was approved by the Research Ethics Committee of the National Taiwan University Hospital (NTUH; reference number: 202003106RINC) and granted a consent waiver. The study results are reported according to the Checklist for Artificial Intelligence in Medical Imaging (CLAIM) [14].

### Image Acquisition and Dataset Designation

**Fig. 1:**
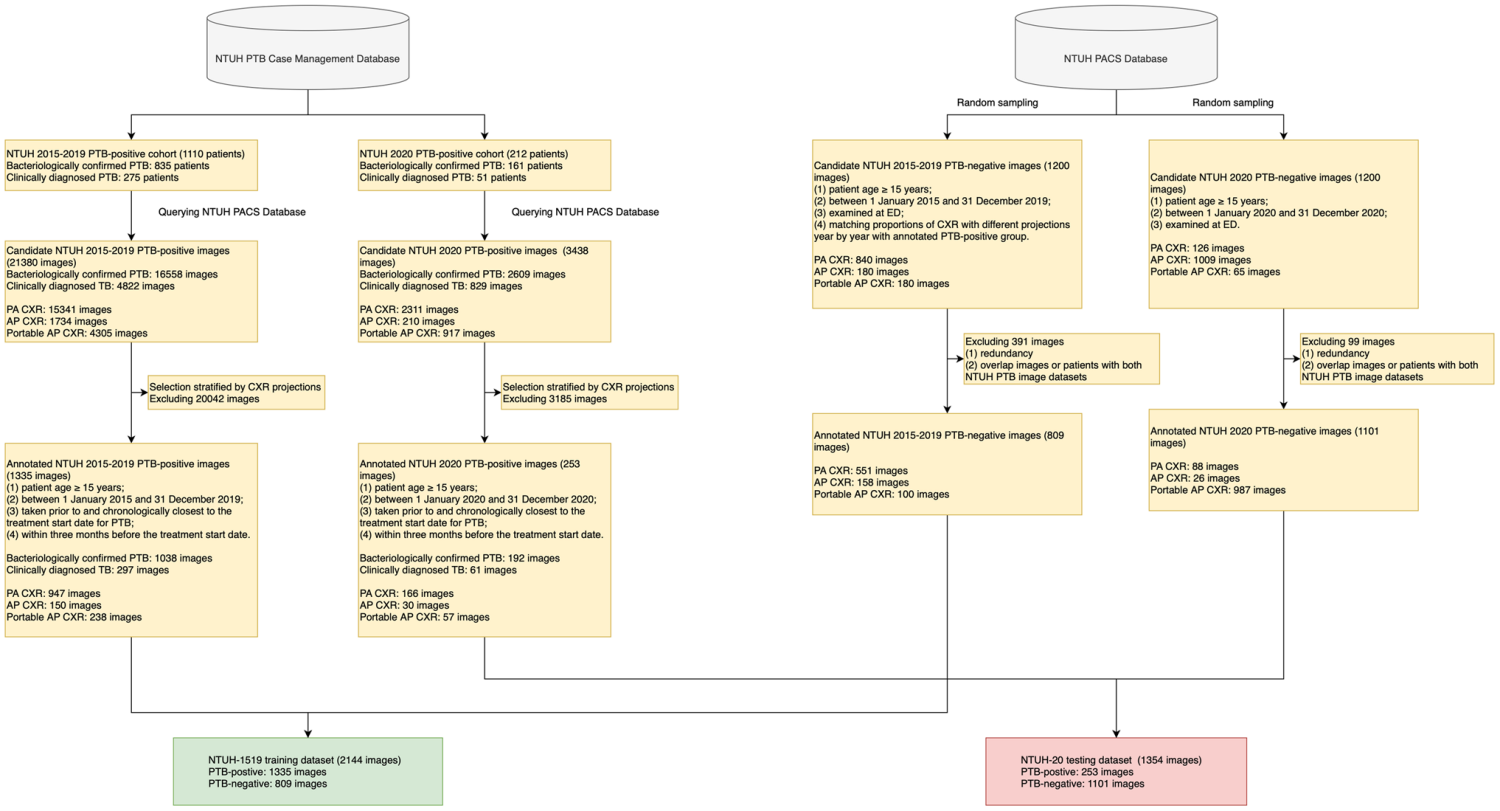
Flowchart of the image inclusion process and dataset designation. CXR, chest X-ray; ED, emergency department; ICU, intensive care unit; NTUH, National Taiwan University Hospital; PACS, Picture Archiving and Communication System

The image acquisition process is shown in Fig. 1. At NTUH, patients diagnosed with PTB are registered in the NTUH PTB Case Management Database and followed prospectively by nurse specialists. The database classifies patients with PTB into two mutually exclusive categories: bacteriologically confirmed PTB or clinically diagnosed PTB [15]. Bacteriologically confirmed PTB was defined as (1) a positive acid-fast bacilli stain (AFS) test along with positive tuberculosis-polymerase chain reaction results in sputum samples or (2) positive sputum culture results for *Mycobacterium tuberculosis*. Clinically diagnosed PTB was diagnosed based on CXR, pathological findings, or other clinical findings, which nonetheless did not fulfil the criteria for bacteriologically confirmed PTB. Candidate lists of patients diagnosed with PTB were retrieved from the Case Management Database and used to query the Picture Archiving and Communication System (PACS) database for candidate CXRs.

Subsequently, the following inclusion criteria were applied to the candidate PTB-positive CXR images to obtain annotated images: (1) patient age ≥ 15 years; (2) examined between 1 January 2015 and 31 December 2020; (3) taken prior to and chronologically closest to the treatment start date for PTB; (4) within 3 months before the treatment start date. These criteria were respectively applied to CXRs filmed in different projections, including posterior-anterior (PA), anterior–posterior (AP), and portable AP CXRs. For comparison cases, to simulate an ED setting [16], candidate PTB-negative images were acquired by a random sample of CXRs taken in the ED with similar inclusion criteria. In addition, for model training, the proportions of different projections of the candidate PTB-negative images were matched to those of the annotated PTB-positive images, while there was no such matching for the model testing. The candidate PTB-negative lists were further examined to avoid the overlap of patients. That is, for each patient, only one image would be allowed for analysis in each projection type. All eligible de-identified CXR images were exported in Digital Imaging and Communications in Medicine (DICOM) format from the PACS database along with the corresponding texts of the radiologists’ reports. These reports were generated by various radiologists for clinical purposes.

The images acquired from NTUH were split chronologically into NTUH-1519 (years 2015 to 2019; model training) and NTUH-20 (year 2020; model testing) datasets. Training and testing were also performed with external public imaging databases for PTB, including NIH ChestX-ray14 for training [17] and Montgomery County [18] and Shenzhen [18] for external testing.

### Image Annotation and Chest X-Ray Report Extraction

For candidate PTB-positive CXR, images were annotated by image-level labelling according to the PTB status registered in the Case Management Database. Both bacteriologically confirmed PTB and clinically diagnosed PTB [15] were annotated as PTB-positive. Candidate PTB-negative CXR images were annotated with a PTB-negative label if the patients with these images had not been diagnosed with PTB and registered in the Case Management Database during the image inclusion period. Both Montgomery County [18] and Shenzhen [18] offered image-level labels, which were used accordingly. For CXR images obtained at NTUH, imaging results and diagnoses [19] were manually extracted from the radiologist reports by research assistants who were blinded to the PTB status of the patients. The diagnoses noted in these clinical reports would be compared with those made by the CAD algorithm.

### Selection of the Algorithm

Two prominent methodologies take the lead in medical image analysis and recognition: Transformers and Convolutional Neural Networks (CNNs). Regarding CNNs, numerous models are available for exploration. For example, Huang et al. [20, 21] employed DenseNet 121 to forge FABNet. Furthermore, Huang et al. [22] extended the utility of FABNet within domain-adaptive tasks, demonstrating the adaptability of these models. Additionally, Zhou et al. [23] highlighted the ability of CNNs to acquire meaningful deep features by utilizing ResNet 50 in constructing LPCANet. To harness the power of transfer learning, Huang et al. [24] leveraged pre-trained models from ImageNet, such as DenseNet121, ResNet50v2, Inception v3, and Inception-ResNet.

As for Transformers, such as ViT (Vision Transformer) [25], Huang et al. [20] pioneered the integration of attention mechanisms with ViT through convolution. Pan et al. [26] further advanced the field by introducing adaptive feature fusion, which amalgamated the strengths of attention mechanisms from both CNNs and ViT. Moreover, Zhou et al. [27] capitalized on the synergy between ResNet and ViT, showcasing promising possibilities.

However, compared with CNNs, ViT’s greater parameter numbers demand more computational resources, and it lacks certain intrinsic features, like rotation and scale invariance, and weight sharing, which can affect its generalization. A recent innovation introduces the Swin Transformer [28], effectively addressing ViT’s computational intensity while demonstrating favourable performance [29].

Our pilot study experimented with different CNNs and the Swin Transformer. Using a subset of the training dataset, NTUH-1519, our pilot study (Supplemental Table 1) demonstrated that the Swin Transformer did not perform as well as the CNN-based algorithms. While the Swin Transformer has shown promise in various contexts, it yielded less favourable results in our specific cases, underscoring the need for ongoing evaluation and adaptability in choosing the most suitable model for specific tasks. According to the pilot study results (Supplemental Table 1), EfficientNetV2 [30] was selected for further model development because of the highest area under the receiver operating characteristic curve (AUC) compared with other algorithms.

### Development of the Algorithm

**Fig. 2:**
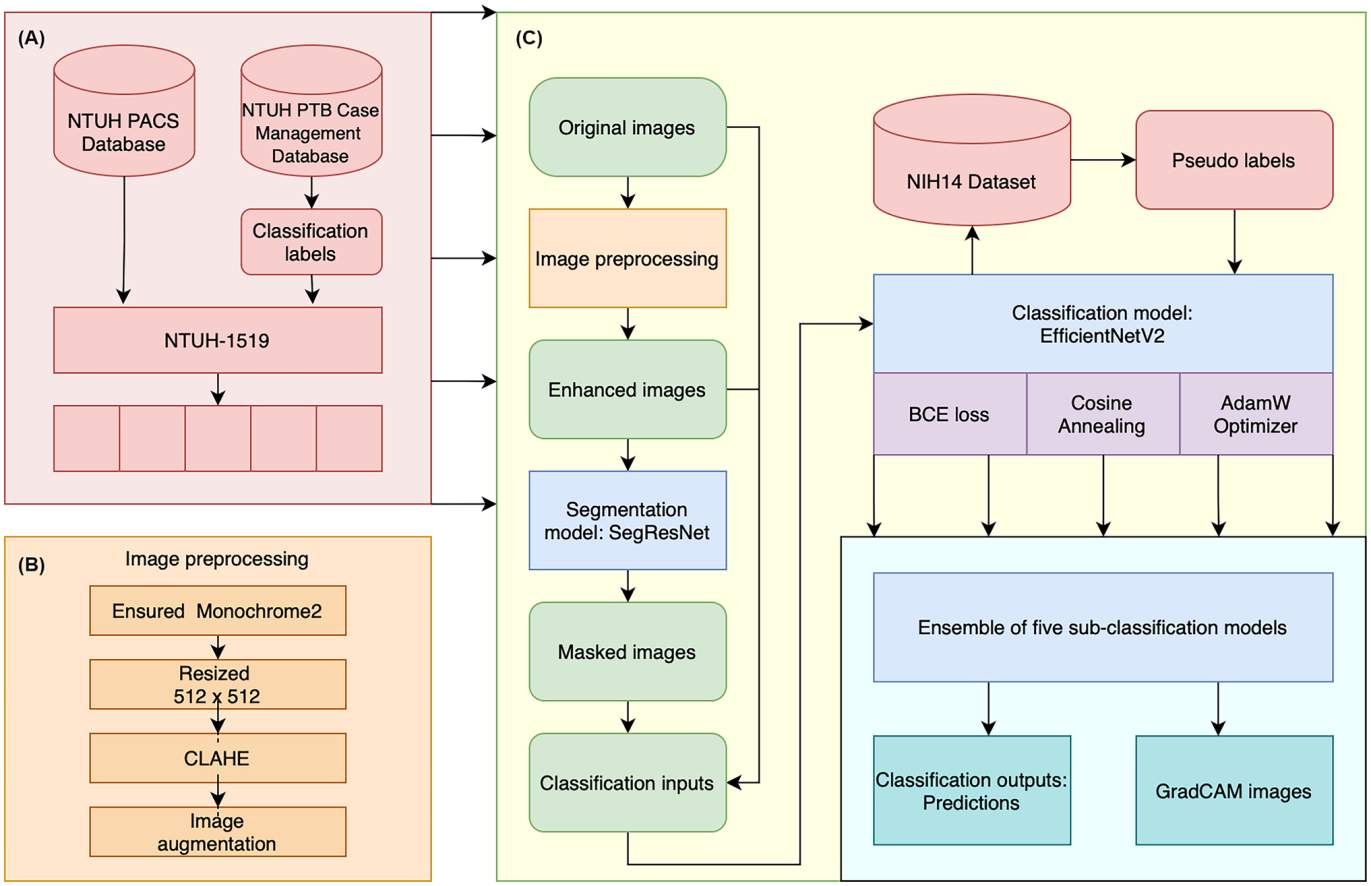
The training pipeline for the CAD algorithm. **A** CXR images were acquired from the NTUH PACS database. The images were annotated by image-level labelling according to the PTB status of the patient (PTB-positive patients were identified from the NTUH PTB Case Management Database). The NTUH-1519 images were randomly split into fivefold for model training. Each fold was used as the validation subset in turn, with other folds as training subsets, to derive five subclassification models for the final ensemble. **B** The original images underwent preprocessing, including confirmation of Monochrome2 output, resizing to 512 × 512 pixels, transformation by contrast limited adaptive histogram equalisation (CLAHE), and image augmentation including horizontal flipping, contrast gaussian noise, and rotate, shear, translate, and scale with zero padding. **C** After preprocessing, the enhanced images were further passed into the segmentation model with SegResNet as the model architecture. The segmentation model segmented out the lung regions which were overlaid on the original images to obtain the masked images. Later, the original, enhanced, and masked images were concatenated and used as input for the classification model. The EfficientNetV2 was adopted as the basic architecture of the classification model. The binary cross entropy loss function was used to supervise the learning process. The training algorithm used AdamW as an optimiser and cosine annealing as a learning rate scheduler. Moreover, we employed a pseudo-labelling method to increase the number of images in the training dataset. After training with images from NTUH-1519, the subclassification models were applied to the NIH ChestX-ray14 dataset to generate PTB pseudo labels. The images with pseudo labels were used to retrain the subclassification models to obtain the final model. **D** The results of the five subclassification models were ensembled to output the final prediction results and GradCAM. CAD, computer-aided detection; CLAHE, contrast limited adaptive histogram equalisation; CXR, chest X-ray; PACS, Picture Archiving and Communication System; PTB, pulmonary tuberculosis

As shown in Fig. 2, the training dataset (NTUH-1519) was randomly split at the image level into five subgroups (called folds) with similar numbers of annotated images across different labels for model development. Each fold was used as the validation subset in turn, with other folds as training subsets to derive five sub-classification models for the final ensemble. The concept underlying ensemble learning is that by amalgamating the predictions from multiple models, any weaknesses and errors inherent to individual models can be mitigated through the strengths of others. This approach amplifies the overall model’s reliability and predictive accuracy.

All images underwent preprocessing to enhance the image contrast details, including contrast limited adaptive histogram equalisation (CLAHE) [31]. SegResNet [32] was used to segment out lung regions to obtain masked images. Then, the original, enhanced, and masked images were used as input for the classification model, for which EfficientNetV2 [30] with binary cross entropy (BCE) was the basic architecture. During the model training process, the batch size was 16, the learning rate was 5e^−5^, and the AdamW optimiser was used. A BCE loss function was used to supervise the learning process. The training procedure was stopped when it reached 20 epochs.

Moreover, we employed a pseudo-labelling method [33] to increase the available labelled images. Pseudo-labelling is a semi-supervised machine learning technique where unlabeled data is assigned predicted labels from a trained model, effectively expanding the training dataset and improving model performance. Following the training with the images from NTUH-1519, the five sub-classification models were respectively applied to the NIH ChestX-ray14 dataset [17], an open dataset containing 112,120 CXRs, to produce PTB pseudo labels. The images with pseudo labels were then used to retrain each sub-classification model to obtain the final model. The predicted probabilities of the five sub-classification models were averaged to make the ensembled prediction, used as the final output of the CAD algorithm. Gradient-weighted class activation mapping (GradCAM) [34] was created to inspect the areas of the image that were activated by the network.

The model was trained on operating system Ubuntu 20.04.4 LTS loaded with the PyTorch 1.10.2 deep learning framework [35], with CUDA 11.6. The training used four Intel^®^ Xeon^®^ CPU E5-2650 v4 at 2.20 GHz processors, 128 GB hard disk space, 16 GB RAM, and a Tesla P100-PCIE-16 GB graphics processing unit (Nvidia Corporation, Santa Clara, CA).

### Evaluation Metrics of the Algorithm

The diagnostic performance was assessed by the AUC, sensitivity, specificity, positive predictive value, and negative predictive value. These evaluation metrics were reported at a threshold selected according to the Youden’s index [36] (CAD algorithm) and also at a threshold established to meet the World Health Organization (WHO) target product profile (TPP) [37] recommendation for a triage tool with at least 90% sensitivity (CAD algorithm: WHO).

### Statistical Analysis

Continuous variables are presented with mean and standard deviation, and categorical variables are presented with counts and proportions. Continuous variables were compared with Student’s *t*-test or ANOVA test, as appropriate. Categorical variables were compared with the chi-squared test. The pair-wise comparison in AUC was performed by the DeLong test [38]. All statistics were expressed with point estimates with 95% confidence intervals (CIs) by a bootstrap technique with 1000 repetitions. Subgroup analysis was performed to explore the influence of patient characteristics and image projections on model performance, and sensitivity analysis was performed to evaluate the diagnostic performance in detecting bacteriologically confirmed PTB. All statistical analyses were carried out by using R 3.4.3.

## Results

### Baseline Characteristics

A total of 3498 images were acquired from the NTUH PACS database, including 2144 images for training (NTUH-1519) and 1354 images for testing (NTUH-20) (Fig. 1). There were significant differences between the NTUH-1519 and NTUH-20 groups, particularly for CXR projections and distribution of types of PTB diagnosis (Table 1). The prevalence of radiologically diagnosed PTB was 0.8% in NTUH-1519 and 0.1% in NTUH-20 (Supplemental Tables 2 and 3).

**Table 1:** Comparisons between the training dataset (NTUH-1519) and the testing dataset (NTUH-20)

Variables	NTUH-1519 images (n = 2144)	NTUH-2020 images (n = 1354)	*p* value
Patient number, *n*	1812	1285	NA
Age, year	62.3 (18.9) (*n* = 1812)	59.5 (19.9) (*n* = 1285)	< 0.001
Male, *n*	1080 (59.6) (*n* = 1812)	691 (53.8) (*n* = 1285)	0.001
Age ≥ 65, *n*	921 (50.8) (*n* = 1812)	594 (46.2) (*n* = 1285)	0.01
CXR projections, *n*			< 0.001
PA view	1498 (69.9)	254 (18.8)	
AP view	308 (14.4)	56 (4.1)	
Portable AP view	338 (15.7)	1044 (77.1)	
Diagnosis of radiologist clinical report, *n*			
PTB	17 (0.8)	2 (0.1)	0.01
Malignancy	13 (0.6)	4 (0.3)	0.20
Pneumonia	52 (2.4)	12 (0.9)	< 0.001
Pneumothorax	54 (2.5)	7 (0.5)	< 0.001
Qualitative descriptive findings in the radiologist clinical report, *n*			
Atelectasis	78 (3.6)	36 (2.7)	0.11
Bronchiectasis	28 (1.3)	6 (0.4)	0.01
Cardiomegaly	497 (23.2)	342 (25.3)	0.16
Cavitation	22 (1.0)	6 (0.4)	0.06
Consolidation	283 (13.2)	109 (8.1)	< 0.001
Emphysema	35 (1.6)	7 (0.5)	0.003
Haziness	196 (9.1)	80 (5.9)	< 0.001
Infiltration	329 (15.3)	255 (18.3)	0.007
Pulmonary oedema	7 (0.3)	2 (0.1)	0.31
Nodule	238 (11.1)	83 (6.1)	< 0.001
Opacification	908 (42.4)	508 (37.5)	0.005
Pleural effusion	516 (24.1)	263 (19.4)	0.001
Annotation, n			< 0.001
PTB-positive	1335 (62.3)	253 (18.7)	
Bacteriologically confirmed PTB	1038 (48.4)	192 (14.2)	
Clinically diagnosed PTB	297 (13.9)	61 (4.5)	
PTB-negative	809 (37.7)	1101 (81.3)	

Data are presented as mean (standard deviation) or counts (proportion)

*NTUH* National Taiwan University Hospital, *PA* posteroanterior, *AP* anteroposterior, *PTB* pulmonary tuberculosis

### Primary Analysis

**Fig. 3:**
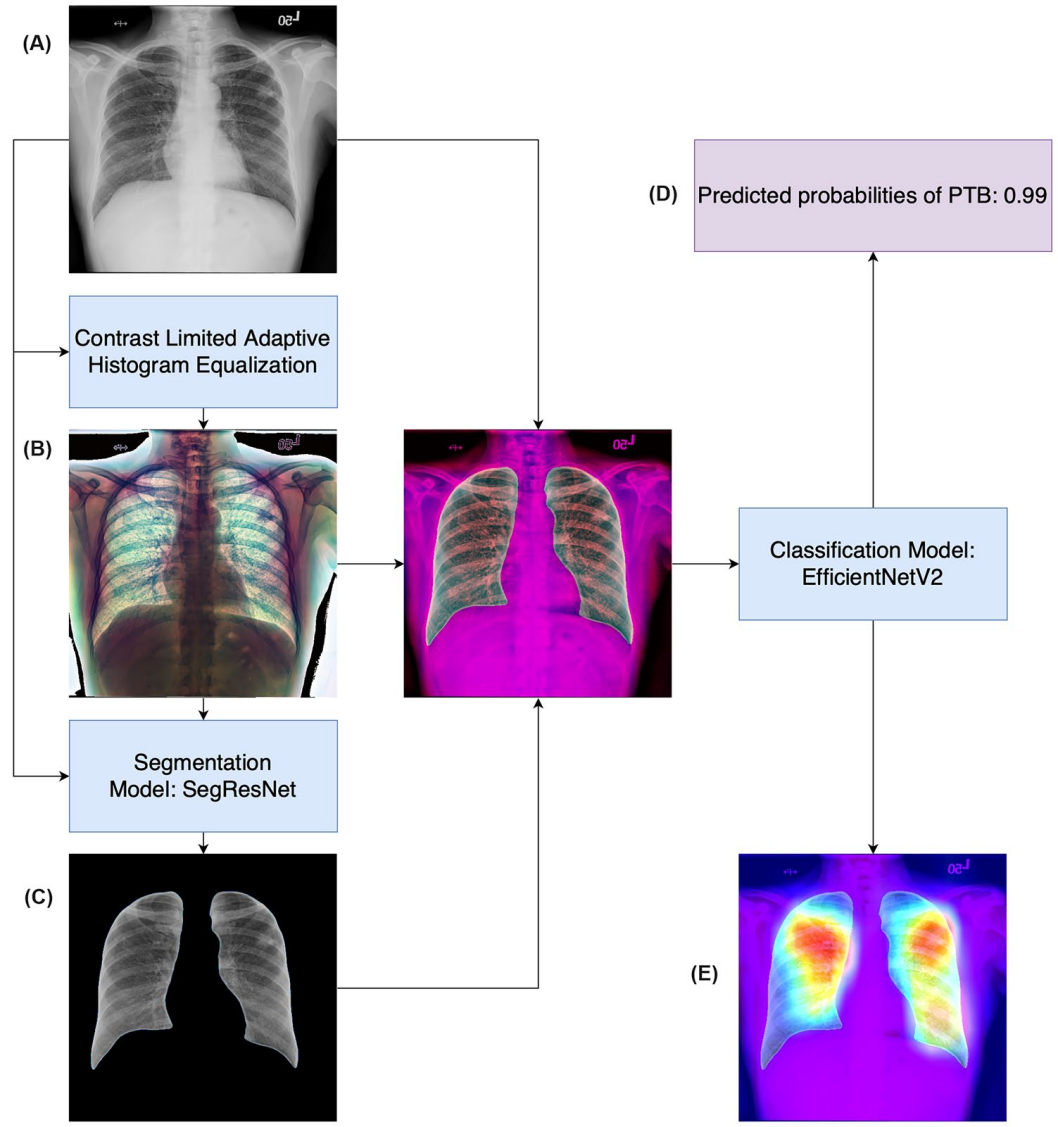
The implementation pipeline of the CAD algorithm. The input of the original CXR image **A** was transformed by CLAHE to obtain the enhanced image **B**. The enhanced image was also passed into the segmentation model to segment the lung regions for the masked images **C**. Subsequently, the original, enhanced, and masked images were concatenated and fed into the classification model to obtain the predicted probabilities for the presence of PTB **D** and for the gradient-weighted class activation mapping (Grad-CAM) (E). We chose SegResNet as the basic architecture of the segmentation model and EfficientNetV2 as the basic architecture for the classification model. CAD, computer-aided detection; CLAHE, contrast limited adaptive histogram equalisation; CXR, chest X-ray; PTB, pulmonary tuberculosis

**Fig. 4:**
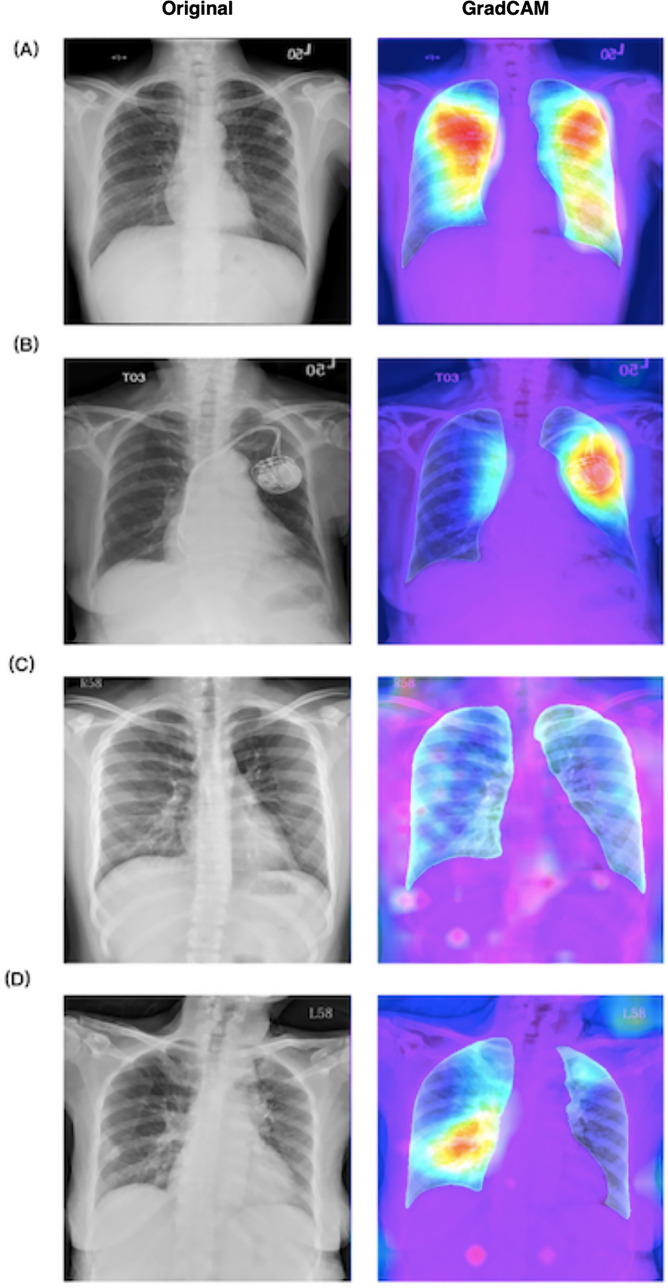
Representative images stratified by predicted results of the computer-aided diagnosis algorithm, including **A** true positive, **B** false positive, **C** true negative, and **D** false negative results for pulmonary tuberculosis. The left column presents the original images. The right column shows the results of gradient-weighted class activation mapping (GradCAM) demonstrating that the algorithm mainly made inferences by using the areas within the segmented lung regions. The reddish regions in the GradCAM represent higher predicted probabilities of pulmonary tuberculosis and the bluish regions represent lower probabilities

A simplified flowchart for implementation of the algorithm is presented in Fig. 3, and four sets of representative images stratified by the prediction results of the algorithm are presented in Fig. 4. The GradCAM indicated that the algorithm mainly detected PTB based on the lung regions rather than other irrelevant areas. The CAD algorithm had excellent performance in diagnosing PTB (AUC 0.878, 95% CI 0.854–0.900; sensitivity 0.783, 95% CI 0.733–0.831) in NTUH-20 (Table 2). The AUC of the CAD algorithm was significantly higher than that of the radiologist reports (AUC 0.504, 95% CI 0.500–0.510, *p*-value < 0.001). When the probability threshold was set at 90% sensitivity [37], the CAD algorithm WHO reached a sensitivity of 0.846 (95% CI 0.802–0.890) and a specificity of 0.667 (95% CI 0.638–0.694) in NTUH-20.

**Table 2:** Diagnostic performance of the computer-aided diagnosis algorithm and the radiologist reports

Scenario	Readers	*N*	AUC	*p* value for comparisons in AUC	Sensitivity	Specificity	PPV	NPV
***NTUH-20 testing dataset***								
** Primary analysis**								
Detecting PTB in all images	CAD algorithm	1354	0.878 (0.854–0.900)	Reference	0.783 (0.733–0.831)	0.775 (0.749–0.798)	0.444 (0.395–0.488)	0.939 (0.924–0.953)
Detecting PTB in all images	Radiologists’ clinical reports	1354	0.504 (0.500–0.510)	< 0.001^a^	0.008 (0.000–0.020)	1.000 (1.000–1.000)	1.0 (1.0–nan)	0.814 (0.794–0.835)
Detecting PTB in all images	CAD algorithm: WHO	1354	0.878 (0.854–0.900)	NA	0.846 (0.802–0.890)	0.667 (0.638–0.694)	0.368 (0.33–0.406)	0.95 (0.934–0.965)
** Subgroup analysis**								
* CXR projection*				< 0.001^b^				
Detecting PTB in PA CXR	CAD algorithm	254	0.940 (0.912–0.965)		0.825 (0.762–0.881)	0.875 (0.802–0.941)	0.926 (0.882–0.965)	0.726 (0.636–0.810)
Detecting PTB in AP CXR	CAD algorithm	56	0.782 (0.644–0.897)		0.567 (0.387–0.733)	0.808 (0.645–0.960)	0.773 (0.588–0.950)	0.618 (0.455–0.765)
Detecting PTB in portable AP CXR	CAD algorithm	1044	0.869 (0.814–0.918)		0.772 (0.649–0.881)	0.765 (0.738–0.789)	0.159 (0.118–0.203)	0.983 (0.973–0.992)
* Age group*				0.04^b^				
Detecting PTB in patients aged ≥ 65 years	CAD algorithm	644	0.859 (0.822–0.890)		0.789 (0.724–0.847)	0.715 (0.677–0.757)	0.491 (0.431–0.548)	0.907 (0.876–0.934)
Detecting PTB in patients aged < 65 years	CAD algorithm	710	0.888 (0.849–0.926)		0.770 (0.684–0.859)	0.77 (0.684– 0.859)	0.77 (0.684– 0.859)	0.77 (0.684– 0.859)
* Sex group*				0.95^b^				
Detecting PTB in male patients	CAD algorithm	737	0.876 (0.843–0.906)		0.800 (0.738–0.857)	0.748 (0.714–0.784)	0.478 (0.419–0.535)	0.928 (0.905–0.950)
Detecting PTB in female patients	CAD algorithm	616	0.875 (0.833–0.912)		0.750 (0.657–0.845)	0.803 (0.767–0.835)	0.388 (0.317–0.458)	0.951 (0.931–0.971)
** Sensitivity analysis**								
Detecting bacteriologically confirmed PTB	CAD algorithm	1354	0.854 (0.823–0.883)	0.04^c^	0.781 (0.721–0.840)	0.745 (0.718–0.770)	0.336 (0.290–0.379)	0.954 (0.940–0.966)
***Montgomery County public dataset***								
Detecting PTB in all images	CAD algorithm	138	0.838 (0.765–0.904)	0.03^d^	0.948 (0.886–1.000)	0.350 (0.250–0.457)	0.514 (0.417–0.610)	0.903 (0.789–1.000)
***Shenzhen public dataset***								
Detecting PTB in all images	CAD algorithm	662	0.806 (0.771–0.839)	0.03^d^	0.188 (0.145–0.229)	1.000 (1.000–1.000)	1.000 (1.000–1.000)	0.544 (0.506–0.585)

Data are presented as point estimates (95% confidence interval). CAD algorithm: the probability threshold for PTB was set at Youden’s index in NTUH-1519. CAD algorithm: WHO indicates the algorithm was adjusted to diagnose PTB at the threshold of 90% sensitivity in NTUH-1519

*AP* anteroposterior, *AUC* area under the receiver operating characteristics curve, *CAD* computer-aided diagnosis, *CXR* chest X-ray, *NA* not applicable, *NPV* negative predictive value, *NTUH* National Taiwan University Hospital, *PA* posteroanterior, *PPV* positive predictive value, *PTB* pulmonary tuberculosis, *WHO* World Health Organization

^a^The comparison was made between the CAD algorithm (reference) and radiologists’ clinical reports by DeLong test

^b^The comparison was made between the subgroups stratified by CXR projection, age or sex by ANOVA or Student’s *t*-test

^c^The comparison was made when the CAD algorithm was applied to detect PTB (reference) versus bacteriologically confirmed PTB

^d^The comparison was made when the CAD algorithm was tested in the NTUH-20 testing dataset (reference) versus Montgomery County public dataset or Shenzhen public dataset

### Subgroup and Sensitivity Analyses

In the subgroup analysis, the CAD algorithm had the best performance in the PA views (AUC 0.940, 95% CI 0.912–0.965,* p*-value < 0.001) compared with AP (AUC 0.782, 95% CI 0.644–0.897) or portable AP views (AUC 0.869, 95% CI 0.814–0.918) (Table 2). The CAD algorithm could detect PTB more accurately in patients aged < 65 years (AUC 0.888, 95% CI 0.849–0.926,* p*-value = 0.04) than ≥ 65 years (AUC 0.859, 95% CI 0.822–0.890). In contrast, no significantly different performance of the CAD algorithm was noted between male and female patients. The sensitivity analysis demonstrated that the CAD algorithm also had excellent performance in distinguishing bacteriologically confirmed PTB (AUC 0.854, 95% CI 0.823–0.883).

### Validation in the External Datasets

Finally, the CAD algorithm was also tested with good performance in the Montgomery County (AUC 0.838, 95% CI 0.765–0.904) and Shenzhen (AUC 0.806, 95% CI 0.771–0.839) databases.

## Discussion

### Main Findings

EfficientNetV2 [30] was adopted in our study, which had shown superior efficiency in previous studies [39, 40]. Our CAD algorithm’s performance was further augmented through an ensemble [41], which was expected to prevent the algorithm from overfitting on a small dataset, thus improving its potential for external generalizability.

### Pulmonary Tuberculosis-Positive Images

Many PTB-detecting algorithms are subject to a high risk of bias because a diagnosis made by human readers is adopted as the reference standard [3]. A derived algorithm which uses human readers as the gold standard may miss many PTB patients. Only 50.5% of ED patients with PTB had “typical” CXR findings [42] and atypical presentations on CXR were found in 63% of patients with delayed isolation in the ED [43]. Among the PTB-positive images in our study, only a minor proportion were diagnosed by CXR (Supplemental Tables 2 and 3). Radiological reports showed only 0.8% sensitivity for PTB in NTUH-20 (Table 2), and this was similar to previous studies [44]. This apparently suboptimal performance of human readers is mostly a function of the broad differential diagnosis clinicians must consider before arriving at the definitive diagnosis [45], and because of this, using human readers as the reference standard creates a risk of systematic overestimation of the diagnostic accuracy of the CAD algorithms [3].

Besides human readers, most other studies [3] have used bacteriologically confirmed PTB as the target. To the best of our knowledge, our CAD algorithm may be the first to detect both bacteriologically confirmed and clinically diagnosed PTB. As there were some overlaps in CXR findings between these two types of PTB [46], the only way to differentiate one from the other is by collecting specimens for examination, such as a sputum smear. Since timely isolation and prompt examination is necessary for patients with both types of PTB diagnosis, we selected both as the target labels in our study. The sensitivity analysis exhibited that the CAD algorithm was able to distinguish bacteriologically confirmed PTB (AUC: 0.854) with excellent performance. This should be reassuring because bacteriologically confirmed PTB is generally considered more infectious than clinically diagnosed PTB.

### Pulmonary Tuberculosis-Negative Images

Most studies [3] have developed and tested PTB-detecting algorithms using popular public databases. As with most public databases, Montgomery County [18] and Shenzhen [18] use normal CXR images as the PTB-negative images. In contrast, in our study, as the PTB-negative images were acquired through a random sampling of CXRs obtained from the ED, there were various pathological radiological findings even in the PTB-negative images (Supplemental Tables 2 and 3). Since PTB has few pathognomonic radiological features, it may be inherently difficult for the algorithm, as it is for human readers, to distinguish between PTB and other look-alike diseases including cancer or pneumonia. The difference in our method of selecting PTB-negative images may partly explain why the AUC for our CAD algorithm was not as high as the AUCs previously reported for algorithms trained by using the public databases [3]. As there might be apparent differences between PTB-positive and normal CXRs, the performance of previous algorithms might be overestimated [3].

In our study, there may be a concern that these PTB-negative patients might have had PTB but been left undiagnosed. This kind of misclassification bias may increase the false positive rate and decrease the AUC of the algorithm. That there were no radiologists’ diagnoses of PTB among the PTB-negative images in NTUH-20 (Supplemental Table 3) may mitigate this bias to some degree.

### External Testing, Subgroup, and Sensitivity Analysis

Our CAD algorithm was tested in a temporally split local dataset, i.e. NTUH-20. As recommended by the TRIPOD statement [47], this type of splitting can be regarded as a type of external testing, as evidenced by the significant differences between NTUH-1519 and NTUH-20 (Table 1). Especially for CXR projections, portable AP CXR was the predominant type of projection in NTUH-20, whereas in NTUH-1519, PA CXR was the dominant projection type (Table 1). For NTUH-1519, matching the proportions of projections was assumed to facilitate the CAD algorithm in learning features of PTB without being biased by the projections. In contrast, a random sample without matching in the NTUH-20 may be more likely to test the CAD algorithm by simulated ED data.

Our CAD algorithm had an AUC of 0.878 when tested in NTUH-20 (Table 2). This is comparable to other algorithms [48]. Our CAD algorithm: WHO displayed a sensitivity of 0.846 and a specificity of 0.667. This is slightly lower than the WHO-recommended minimum requirement of > 90% sensitivity and > 70% specificity for a PTB triage tool [37]. However, these WHO requirements are indicated for patients with any symptoms or risk factors for active PTB. This group probably has a different prevalence of PTB than the variety of patients included in NTUH-20, who would likely have presented to ED with all kinds of symptoms. That the AUCs of our CAD algorithm were similar in NTUH-20 (0.878), Montgomery County (0.838), and Shenzhen (0.806) highlights the favourable potential of our CAD algorithm for external generalizability. Interestingly, the sensitivity and specificity of our CAD algorithm were balanced in NTUH-20 while inclined to high sensitivity in Montgomery County and high specificity in Shenzhen. As there may be substantial differences in the PTB burden in different clinical scenarios, adjusting the threshold of the CAD algorithm to reflect PTB prevalence in the local population is recommended [49].

Most studies [3] have adopted PA CXR to derive an algorithm because PA CXR is considered the gold standard in plain chest radiography. AP or even portable AP CXR is considered suboptimal for diagnosis. As demonstrated in our subgroup analysis, the performance of the CAD algorithm was significantly higher (AUC 0.940) in the PA CXR projections. The subgroup analysis results may explain the lower AUC of our CAD algorithm compared with other studies using PA CXR [3]. Also, this result may suggest that the PA CXR-derived algorithm should not be directly applied to AP or portable AP CXR images for PTB detection. Finally, as suggested by previous studies [48, 49], our subgroup analysis indicated that the performance of the CAD algorithms would vary by age but not by sex.

### Study Setting and Application in the Emergency Department

Other algorithms [49] have been developed for PTB triage or screening in a referral centre or an area of high prevalence. These algorithms enrolled patients with specific symptoms, such as fever and cough, suggestive of PTB, to test the algorithms [49]. However, it is reported [50] that among ED patients with active PTB, approximately half present with nonspecific symptoms such as abdominal pain [50]. Since our study did not use any clinical information to select the images and used random samples of ED patients as PTB-negative images, our CAD algorithm may be more readily applicable to ED settings. By alerting clinicians who may not have included PTB in their differential diagnosis, the CAD algorithm may reduce the number of missed PTB cases in the ED or shorten the interval between an affected patient’s arrival and airborne isolation.

### Study Limitations

This was a case–control study. The selection method for PTB-negative images may have influenced the algorithm’s performance. Nonetheless, in light of the report that among another cohort of 31,267 consecutive ED visits, only 30 patients (0.1%) were diagnosed with PTB [16]; a case–control study design may be a more efficient way to develop the CAD algorithm. Further prospective studies are warranted to enrol consecutive patients visiting the ED to test the performance in a scalable manner.

## Conclusions

Based on EfficientNetV2, a CAD algorithm can detect PTB on CXR in a simulated ED setting with an AUC of 0.878. The algorithm detected PTB better in the PA than AP or portable AP views. The algorithm can also distinguish bacteriologically confirmed PTB with an AUC of 0.854. Finally, the CAD algorithm also demonstrated good performance in the external datasets, including Montgomery County (AUC 0.838) and Shenzhen (AUC 0.806) databases.

### Supplementary Information

Below is the link to the electronic supplementary material.

Supplementary file1 (DOCX 16 KB)

Supplementary file2 (DOCX 17 KB)

Supplementary file3 (DOCX 17 KB)

## Data Availability

The data that support the findings of this study are available from the corresponding author upon reasonable request.

## Declarations

### Disclaimer

The National Taiwan University Hospital and National Science and Technology Council had no involvement in designing the study, collecting, analysing or interpreting the data, writing the manuscript, or deciding whether to submit the manuscript for publication.
