# Supplementary material for: Deep Learning–based Diagnosis of Pulmonary Tuberculosis on Chest X-ray in the Emergency Department: A Retrospective Study
Source: J Imaging Inform Med. 2024 Jan 10;37(2):589–600. doi: 10.1007/s10278-023-00952-4 (PMC11031502; doi:10.1007/s10278-023-00952-4)
Supplement: Supplementary file 1 — Supplementary file1 (DOCX 16 KB) [file 10278_2023_952_MOESM1_ESM.docx]

**Supplemental Table 1. The results of the pilot study comparing different algorithms**

| Algorithm | AUC | Accuracy | F1 score | Specificity | Sensitivity | Negative predictive value | Positive predictive value |
| --- | --- | --- | --- | --- | --- | --- | --- |
| *Convolutional Neural Network-based algorithms* | | | | | | | |
| Vgg16_bn | 0.920 | 0.819 | 0.812 | 0.770 | 0.880 | 0.890 | 0.753 |
| Inception_v4 | 0.924 | 0.827 | 0.819 | 0.785 | 0.880 | 0.892 | 0.765 |
| Densenetblur121d | 0.906 | 0.786 | 0.792 | 0.679 | 0.921 | 0.915 | 0.695 |
| Efficientnetv2_rw_s | 0.936 | 0.842 | 0.835 | 0.794 | 0.904 | 0.910 | 0.777 |
| *Transformer-based algorithm* |  |  |  |  |  |  |  |
| Swin_base_patch4_window12_384_in22k | 0.898 | 0.822 | 0.785 | 0.863 | 0.767 | 0.835 | 0.804 |
