# Supplementary material for: Deep Learning–based Diagnosis of Pulmonary Tuberculosis on Chest X-ray in the Emergency Department: A Retrospective Study
Source: J Imaging Inform Med. 2024 Jan 10;37(2):589–600. doi: 10.1007/s10278-023-00952-4 (PMC11031502; doi:10.1007/s10278-023-00952-4)
Supplement: Supplementary file 2 — Supplementary file2 (DOCX 17 KB) [file 10278_2023_952_MOESM2_ESM.docx]

Supplemental Table 2. Comparisons between images annotated with and without pulmonary tuberculosis in the XXXH-1519 training dataset

| Variables | PTB-positive images (n=1335) | PTB-negative images (n=809) | *p* value |
| --- | --- | --- | --- |
| Patient number, n | 1008 | 804 | NA |
| Age, year | 63.3 (17.9) (n=1008) | 61.1 (20.1) (n=804) | 0.01 |
| Male, n | 648 (64.3) (n=1008) | 432 (53.7) (n=804) | <0.001 |
| Age ≥65, n | 529 (52.5) (n=1008) | 392 (48.8) (n=804) | 0.12 |
| CXR projections, n |  |  | <0.001 |
| PA view | 947 (70.9) | 551 (68.1) |  |
| AP view | 153 (11.5) | 158 (19.5) |  |
| Portable AP view | 235 (17.6) | 100 (12.4) |  |
| Diagnosis of radiologist report, n |  |  |  |
| PTB | 17 (1.3) | 0 (0) | 0.001 |
| Malignancy | 11 (0.8) | 2 (0.2) | 0.10 |
| Pneumonia | 41 (3.1) | 11 (1.4) | 0.01 |
| Pneumothorax | 44 (3.3) | 10 (1.2) | 0.003 |
| Qualitative descriptive findings in the radiologist report, n |  |  |  |
| Atelectasis | 59 (4.4) | 19 (2.3) | 0.01 |
| Bronchiectasis | 25 (1.9) | 3 (0.4) | 0.003 |
| Cardiomegaly | 259 (19.4) | 238 (29.4) | <0.001 |
| Cavitation | 22 (1.6) | 0 (0) | <0.001 |
| Consolidation | 221 (16.6) | 62 (7.7) | <0.001 |
| Emphysema | 29 (2.2) | 6 (0.7) | 0.01 |
| Haziness | 117 (8.8) | 79 (9.8) | 0.44 |
| Infiltration | 203 (15.2) | 126 (15.6) | 0.82 |
| Lung oedema | 1 (0.07) | 6 (0.7) | 0.009 |
| Nodule | 195 (14.6) | 43 (5.3) | <0.001 |
| Opacification | 711 (53.3) | 197 (24.4) | <0.001 |
| Pleural effusion | 375 (28.1) | 141 (17.4) | <0.001 |

Data are presented as mean (standard deviation) or counts (proportion). XXXH: XXX Hospital; PA: posteroanterior; AP: anteroposterior; PTB: pulmonary tuberculosis
