# Supplementary material for: Deep Learning–based Diagnosis of Pulmonary Tuberculosis on Chest X-ray in the Emergency Department: A Retrospective Study
Source: J Imaging Inform Med. 2024 Jan 10;37(2):589–600. doi: 10.1007/s10278-023-00952-4 (PMC11031502; doi:10.1007/s10278-023-00952-4)
Supplement: Supplementary file 3 — Supplementary file3 (DOCX 17 KB) [file 10278_2023_952_MOESM3_ESM.docx]

Supplemental Table 3. Comparisons between images annotated with and without pulmonary tuberculosis in the XXXH-20 testing dataset

| Variables | PTB-positive images (n=253) | PTB-negative images (n=1101) | *p* value |
| --- | --- | --- | --- |
| Patient number, n | 184 | 1101 |  |
| Age, year | 66.9 (16.7) (n=184) | 58.3 (20.1) (n=1101) | <0.001 |
| Male, n | 119 (64.7) (n=184) | 572 (52.0) (n=1101) | 0.001 |
| Age ≥65, n | 116 (63.0) (n=184) | 478 (43.4) (n=1101) | <0.001 |
| CXR projections, n |  |  | <0.001 |
| PA view | 166 (65.6) | 88 (8.0) |  |
| AP view | 30 (11.9) | 26 (2.4) |  |
| Portable AP view | 57 (22.5) | 987 (89.6) |  |
| Diagnosis of radiologist report, n |  |  |  |
| PTB | 2 (0.8) | 0 (0) | 0.003 |
| Malignancy | 1 (0.4) | 3 (0.3) | 0.75 |
| Pneumonia | 5 (2.0) | 7 (0.6) | 0.04 |
| Pneumothorax | 4 (1.6) | 3 (0.3) | 0.009 |
| Qualitative descriptive findings in the radiologist report, n |  |  |  |
| Atelectasis | 5 (2.0) | 31 (2.8) | 0.45 |
| Bronchiectasis | 5 (2.0) | 1 (0.09) | <0.001 |
| Cardiomegaly | 54 (21.3) | 288 (26.2) | 0.11 |
| Cavitation | 5 (2.0) | 1 (0.1) | <0.001 |
| Consolidation | 37 (14.6) | 72 (6.5) | <0.001 |
| Emphysema | 3 (1.2) | 4 (0.4) | 0.10 |
| Haziness | 15 (5.9) | 65 (5.9) | 0.99 |
| Infiltration | 53 (20.9) | 202 (18.3) | 0.34 |
| Lung oedema | 0 (0) | 2 (0.2) | 0.50 |
| Nodule | 36 (14.2) | 47 (4.3) | <0.001 |
| Opacification | 165 (65.2) | 343 (31.2) | <0.001 |
| Pleural effusion | 75 (29.6) | 188 (17.1) | <0.001 |

Data are presented as mean (standard deviation) or counts (proportion). XXXH: XXX Hospital; PA: posteroanterior; AP: anteroposterior; PTB: pulmonary tuberculosis
